# Evidence-based target setting informs blue carbon strategies for nationally determined contributions

**DOI:** 10.1038/s41559-023-02081-1

**Published:** 2023-06-01

**Authors:** Katie K. Arkema, Jade M. S. Delevaux, Jessica M. Silver, Samantha G. Winder, Lisa M. Schile-Beers, Nadia Bood, Stephen Crooks, Karen Douthwaite, Courtney Durham, Peter L. Hawthorne, Thomas Hickey, Colin Mattis, Andria Rosado, Mary Ruckelshaus, Moritz von Unger, Arlene Young

**Affiliations:** 1 Natural Capital Project, Stanford University, Stanford, CA USA; 2 School of Marine and Environmental Affairs, University of Washington, Seattle, WA USA; 3 Pacific Northwest National Laboratory, Seattle, WA USA; 4 School of Environmental and Forest Sciences, University of Washington, Seattle, WA USA; 5 Outdoor Recreation and Data Lab, University of Washington, Seattle, WA USA; 6 Silvestrum Climate Associates, Sausalito, CA USA; 7 World Wildlife Fund Mesoamerica, Belize Field Office, Belize City, Belize; 8 Ocean Conservation, World Wildlife Fund, Washington, DC USA; 9 The Pew Charitable Trusts, Washington, DC USA; 10 Institute on the Environment, University of Minnesota, Saint Paul, MN USA; 11 National Climate Change Office, Belmopan, Belize; 12 Coastal Zone Management Authority and Institute, Belize City, Belize

**Keywords:** Climate-change mitigation, Sustainability, Climate-change adaptation, Climate-change mitigation, Environmental economics

## Abstract

The magnitude and pace of global climate change demand ambitious and effective implementation of nationally determined contributions (NDCs). Nature-based solutions present an efficient approach to achieving mitigation, adaptation and resilience goals. Yet few nations have quantified the diverse benefits of nature-based solutions to evaluate and select ecosystem targets for their NDCs. Here we report on Belize’s pursuit of innovative, evidence-based target setting by accounting for multiple benefits of blue carbon strategies. Through quantification of carbon storage and sequestration and optimization of co-benefits, we explore time-bound targets and prioritize locations for mangrove protection and restoration. We find increases in carbon benefits with larger mangrove investments, while fisheries, tourism and coastal risk-reduction co-benefits grow initially and then plateau. We identify locations, currently lacking protected status, where prioritizing blue carbon strategies would provide the greatest delivery of co-benefits to communities. These findings informed Belize’s updated NDCs to include an additional 12,000 ha of mangrove protection and 4,000 ha of mangrove restoration, respectively, by 2030. Our study serves as an example for the more than 150 other countries that have the opportunity to enhance greenhouse gas sequestration and climate adaptation by incorporating blue carbon strategies that provide multiple societal benefits into their NDCs.

## Abstract (web summary)

An assessment of blue carbon strategies in Belize shows how quantifying fisheries, tourism and coastal risk co-benefits alongside carbon benefits can inform spatial and temporal target setting for nationally determined climate contributions that simultaneously provide societal benefits.

## Main

Global increases in temperature threaten communities and ecosystems around the world. The Intergovernmental Panel on Climate Change (IPCC) special report on warming of 1.5 °C above pre-industrial levels predicts species loss, sea-level rise, flooding and droughts^1^. Such impacts will disproportionately affect disadvantaged and vulnerable populations, especially those highly dependent on natural resources for their livelihoods and those that live in small island developing states and least developed countries^1–3^. To confront the growing magnitude and pace of climate change, it is paramount that countries increase the ambition and improve the implementation of their nationally determined contributions (NDCs)^4,5^. The NDCs describe a set of measures each country aims to take towards achieving the global goal outlined in the Paris Agreement of stabilizing warming ‘well below’ 2 °C and pursuing efforts to limit the temperature increase to 1.5 °C above pre-industrial levels. Given the lack of progress in controlling emissions^6,7^, nations at the 26th United Nations Climate Change Conference of the Parties (COP26) agreed to revisit and strengthen their current NDCs before the next major update in 2025^8^.

Nature-based solutions provide a promising—yet often overlooked—pathway to bolster NDCs. For example, blue carbon strategies involve protecting and sustainably managing coastal and marine ecosystems, which store and sequester carbon and provide a suite of co-benefits that can help communities adapt to climate change^9–11^. Yet 14 coastal parties to the Paris Agreement, including the United States, the European Union, Australia and others, ignored oceans in their initial 2016 NDCs^12^. Although attention to oceans for climate solutions typically lags behind land in international forums^9^, such delays have not hindered efforts in the Central American country of Belize nor in the more than 45 other countries integrating blue carbon strategies into their updated contributions^13,14^. The Belizean government’s pursuit of scientifically robust blue carbon strategies illustrates an innovative approach for evidence-based target setting of nature-based solutions. With the recent refinement of NDCs substantially improving ocean coverage, this approach could be taken up by other countries as they seek to close the emissions gap.

Blue carbon solutions can be a powerful and efficient strategy because they reduce emissions and support adaptation through multiple pathways. Marine ecosystems such as mangroves, seagrasses and salt marshes store and sequester carbon in their sediments, roots and aboveground biomass^15–17^. Estimates suggest that if the annual global coastal wetlands loss was halved, emissions would be reduced by 0.23 Gt CO_2_ yr^−1^, which is comparable to offsetting the 2013 emissions of Spain (0.24 Gt CO_2_ equivalent) annually^18^. Additionally, blue carbon ecosystems sustain fisheries, provide tourism and recreation opportunities, enhance water quality and help to reduce risk due to coastal hazards^2,19–22^. By supporting local livelihoods and protecting shorelines as sea levels rise and storms increase, these co-benefits can enhance the resilience and adaptation of coastal communities to a changing climate^23^.

However, designing specific measures and determining where and how to direct investments to realize climate mitigation and adaptation benefits is a challenge for implementing blue carbon strategies. In contrast to mitigation benefits (which are distributed globally^24^), the magnitude of climate adaptation co-benefits experienced by communities depends on the location of blue carbon ecosystems; fisheries, tourism and coastal risk-reduction outcomes of blue carbon strategies accrue locally and may be distributed unevenly^25–27^. Countries could invest in blue carbon projects in certain locations to support specific priorities, such as climate adaptation of disadvantaged communities^28,29^. But they face potential trade-offs among co-benefits, limited resources and competing interests^4,5,11,14^.

For nature-based solutions, NDCs range from descriptive actions (for example, reduce deforestation) to more quantitative targets, such as the protection of a specified area of wetland or the increase in a country’s carbon sink by a specific amount of CO_2_e through restoration. Formulating effective targets—ones that are measurable, time bound^30^ and address mitigation, adaptation and long-term resilience goals—requires information about the amount of carbon stored and sequestered and estimates of where nature-based strategies would deliver multiple and equitable co-benefits. This information is hard to gather^5,14,31^. Among the original NDCs that included current or planned nature-based solutions, less than one-fifth of nations set robust, quantifiable targets^11^. With current NDCs indicating a sizable increase in global emissions in 2030 and some countries revisiting their contributions this year, there is a need for approaches and analyses that inform choices related to ambition and effective implementation.

To design scientifically rigorous and actionable targets for blue carbon and to prioritize locations for implementation, Belize quantified carbon storage and sequestration and optimized co-benefits provided by mangroves. As a prominent member of the Alliance of Small Island States, Belize has a history of global leadership in ocean sustainability^25,32,33^. However, like many other countries around the world, Belize is weighing how to deliver sustainable economic growth, especially in the wake of the COVID-19 pandemic. Linking blue carbon ecosystems to livelihoods and adaptation has potential to align the country’s economic and climate policies to achieve multiple goals^12,13,31,34^.

**Extended Data Fig. 1:**
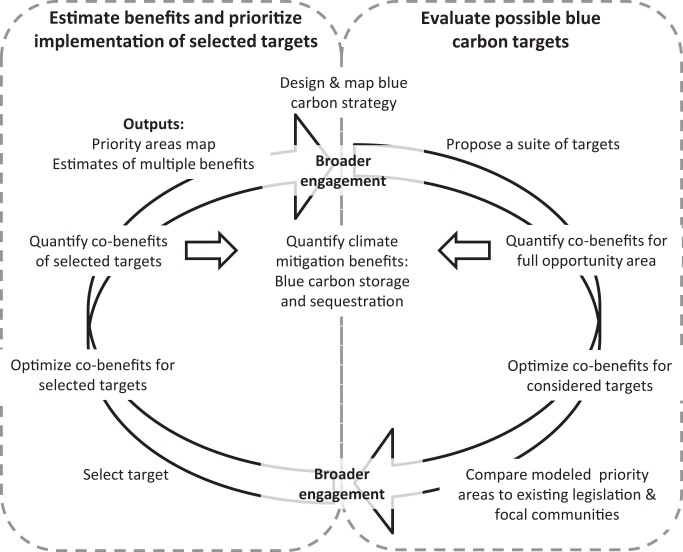
Iterative approach for evaluating and selecting blue carbon targets to inform NDCs. The workflow begins on the right-hand side of the figure with an evaluation of potential blue carbon targets based on quantification of carbon storage and sequestration and optimization of co-benefits. The analytical approach is repeated on the left-hand side of the figure to assess the benefits of selected targets. The entire process is meant to be iterative to inform future updates to a country’s NDCs.

Given these opportunities and challenges, our working group of stakeholders, policymakers and scientists tackled two main questions: (1) what are the carbon mitigation and climate adaptation co-benefits produced by a range of potential blue carbon targets and (2) where should policies and actions be prioritized to maximize a suite of co-benefits? To address these questions, we defined two blue carbon strategies—mangrove protection and restoration—and a suite of possible targets for the extent of these strategies by 2030, the time horizon for Belize’s updated NDCs and national development plan. We quantified carbon storage and sequestration using land-cover data from Belize and existing nearby field estimates from Mexico. We quantified coastal risk reduction, tourism and fisheries co-benefits by modelling ecosystem services provided by mangroves currently and under the two blue carbon strategies (Methods). To identify priority locations for investing in blue carbon strategies, we optimized co-benefits. We conducted two iterations of this analytical approach to first evaluate possible targets and then estimate the benefits and prioritize the implementation of selected targets (Extended Data Fig. 1). By assessing where investments in mangrove protection and restoration would lead to the greatest return in carbon storage and sequestration and tourism, fisheries and coastal risk reduction, this study demonstrates the power of quantifying co-benefits provided by nature-based solutions for climate change to inform the NDCs.

## Results

### Evaluate a suite of possible blue carbon targets

**Extended Data Fig. 2:**
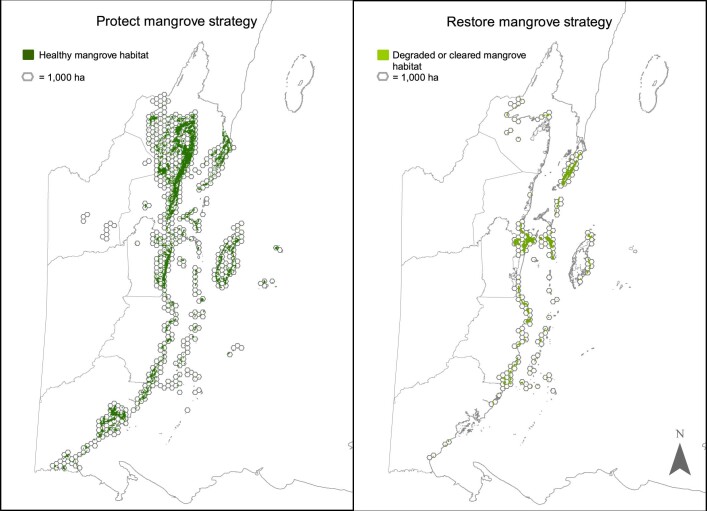
Full opportunity area for blue carbon strategies. Full opportunity area for mangrove protection and mangrove restoration based on location of intact (protect) and degraded or lost (restore) mangroves.

To evaluate potential blue carbon targets, we first mapped the full opportunity area for implementing each of the two strategies and quantified carbon and co-benefits (right side of Extended Data Fig. 1). We identified where ‘protect mangrove’ and ‘restore mangrove’ strategies could be implemented using remotely sensed information on mangrove area, health, degradation and clearing (Methods and Supplementary Table 1). On the basis of these data, we classified mangrove habitat as appropriate for either protection (that is, healthy intact mangroves) or restoration (that is, degraded or cleared mangrove areas; Methods and Extended Data Fig. 2). We then quantified carbon and co-benefits for the two strategies, with the estimates of implementing protection measured against complete loss of mangroves (Methods and Discussion). Our analysis reveals that protecting the remaining 64,000 ha of healthy mangroves across Belize would preserve up to 41.1 million metric tons (MMT) C (150.6 MMT CO_2_e) in total organic carbon stock and carbon dioxide equivalents, respectively. In addition, this would safeguard 800,000 pounds of spiny lobster catch worth $6 million BZD annually, foster continued visitation of at least 4,000 tourists to mangrove destinations annually (generating $600,000 BZD in expenditures) and nearly halve the number of people otherwise at highest risk from coastal hazards in 2030 without risk reduction provided by mangroves. Likewise, restoring 13,000 ha of mangrove would increase total organic carbon stock by 1.67 MMT C (6.12 MMT CO_2_e), augment lobster catch by 700,000 pounds annually, draw an additional 20,000 visitors annually (generating nearly $3 million BZD) and further reduce risk from coastal hazards. Interestingly, the relatively high values of tourism and lobster benefits resulting from restoration (of a smaller area), as compared to protection (of a larger area), suggest that the most valuable mangrove habitat may have already been lost and thus would be well worth the effort to recover. In contrast, total organic carbon stock is lower for restoration than protection because the area restored is smaller and it takes time for carbon stocks to accumulate in the soil and biomass (Methods).

**Fig. 1:**
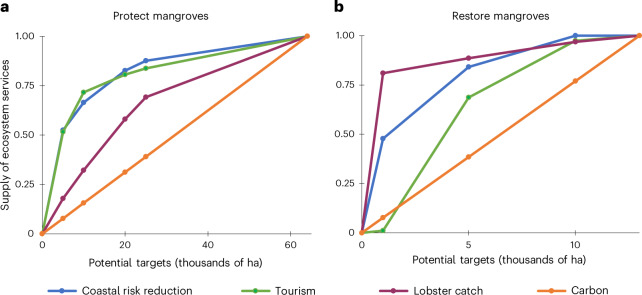
Climate mitigation and co-benefits for potential blue carbon targets. **a**,**b**, Climate mitigation and co-benefits for each potential mangrove protection (**a**) and restoration (**b**) target relative to the benefits provided by the full opportunity area for each strategy. These estimates were calculated using mangrove footprints based on the priority areas selected through optimization of ecosystem services (Fig. 2). Protection includes highest estimates for carbon storage and sequestration because not all mangroves are at risk of degradation currently; restoration includes estimates for carbon sequestration. The *y* axis for **a** represents the supply of ecosystem services attributable to the implementation of this strategy, assuming that without protection, these healthy mangroves would be degraded such that they are no longer functionally able to provide benefits (Methods).

Next we consulted with our working group of stakeholders, policymakers and scientists to identify a range of potential targets for the area of mangroves protected and restored by 2030 (Supplementary Table 2 and Methods), considering the limited capacity in Belize to support existing protected areas and the tremendous difficulties restoration efforts face. We optimized the co-benefits provided by each ecosystem target to identify priority locations for protection and restoration and then estimated carbon, fisheries, tourism and coastal risk-reduction benefits. With greater target areas, we find more carbon storage and sequestration. We also find increasing delivery of coastal risk reduction, tourism and lobster services, with diminishing returns as the upper bound of area protected or restored is approached (Fig. 1). The location of the inflection points—where the rate of increase of each service begins to decrease as mangrove area continues to rise—can help stakeholders and policymakers explore levels of ambition for mitigation and adaptation co-benefits. For mangrove protection, our results suggest that diminishing returns of coastal protection and tourism occur at smaller areas of investment than lobster, perhaps due to the nonlinear benefits of ecosystems for reducing risks from coastal hazards or the concentration of tourists to a few locations. By selecting 12,000 ha (beyond the existing ≈13,000 ha for a total of 25,000 ha) of mangrove protected, Belize achieves a more ambitious carbon storage target and safeguards the most important nursery habitat for lobster per unit area of mangroves. For restoration, the rate of increase in co-benefits begins to flatten by the selected 4,000 ha target amount; however, the potential for further carbon sequestration continues to grow (Methods), suggesting more ambitious targets for mangrove restoration could help to reduce emissions.

**Fig. 2:**
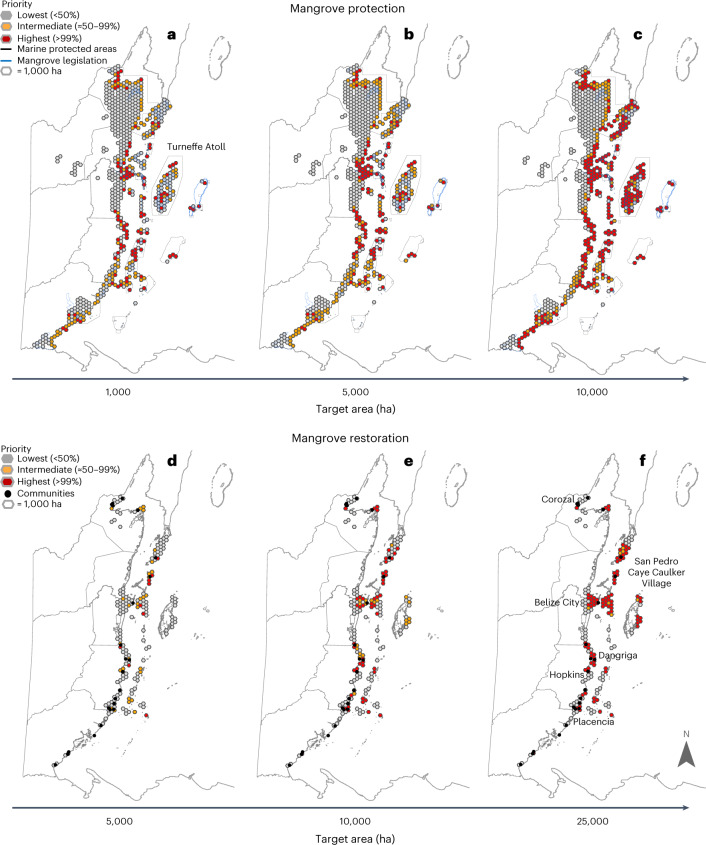
Priority locations for potential blue carbon targets. **a**–**f**, Priority locations for investing in 5,000 ha (**a**), 10,000 ha (**b**) and 25,000 ha (**c**) of mangrove protection (top row) and 1,000 ha (**d**), 5,000 ha (**e**) and 10,000 ha (**f**) of mangrove restoration (bottom row). Priority is based on the number of times a hexagon is selected out of 1,000 model runs in the optimization analysis. Mangrove legislation priorities are the most critical areas for mangrove protection in Belize as designated in recent national mangrove regulation that was based on extensive stakeholder consultation. Communities are the main cities, towns and settlements in Belize where people rely on benefits of mangroves and other coastal ecosystems for their sustenance, livelihoods and coastal climate mitigation and protection.

Our analysis also reveals that not all areas generate equal benefits. Spatial optimization highlights the locations that would provide the greatest, and intermediate, provisioning of multiple ecosystem services (red and orange grid cells in Fig. 2, respectively). By overlaying the priority results with a map of existing protected areas, we highlight important places for investing in mangrove protection (for example, Belize City) that are also outside existing marine protected areas (MPAs) versus places such as Turneffe Atoll that already receive some form of protection for mangroves or where clear-cutting, access or extraction of any kind are prohibited (Fig. 2a–c). Similarly, several priority areas for mangrove restoration are near communities that would benefit from this restoration (for example, Belize City, Corozal, Caye Caulker Village, San Pedro, Dangriga, Hopkins, Placencia), while other priority areas occur further from the people that may need these benefits the most (Fig. 2d–f).

### Assess benefits and prioritize areas for selected targets

Next we estimated benefits and prioritized implementation of blue carbon targets adopted by the government of Belize in its updated NDC (left side of Extended Data Fig. 1). According to results from the ecosystem service models, protecting 12,000 ha of currently unprotected mangroves by 2030 would preserve a total carbon stock of up to 7.7 MMT C (28.4 MMT CO_2_e), safeguard 300,000 pounds of spiny lobster catch worth $2.5 million BZD annually, foster continued visitation of at least 3,000 tourists to mangrove destinations (resulting in $800,000 BZD in tourism expenditures annually) and reduce by more than a third the population that otherwise would be at highest risk to coastal hazards without mangrove protection. Fulfilment of the 4,000 ha restoration target would capture an additional 0.48 MMT C (1.77 MMT CO_2_e), increase the delivery of lobster catch by 600,000 pounds annually, draw an additional 11,000 visitors and further reduce risk from coastal hazards.

In some cases, these absolute changes in benefits are substantial. According to our analysis, mangrove restoration could increase lobster catch by about two-thirds annually, while protection supports long-term resilience of local populations. On the other hand, tourism benefits are less sizable relative to total coastal tourism (≈1.2 million people annually), as visitors come to Belize for many reasons besides mangroves. For carbon, 100% of the restoration benefits are considered climate mitigation benefits. In contrast, only about two-thirds of the areas for protection are at risk from cumulative effects of coastal activities and stressors^35,36^ and thus could be counted towards carbon mitigation. Moreover, the Belize NDC takes a conservative approach, noting that although mangrove protection is included in both its mitigation and adaptation targets, ‘protection is a non-CO_2_e commitment because mangrove loss has been negligible over the last 20 years’ (ref. ^13^).

**Fig. 3:**
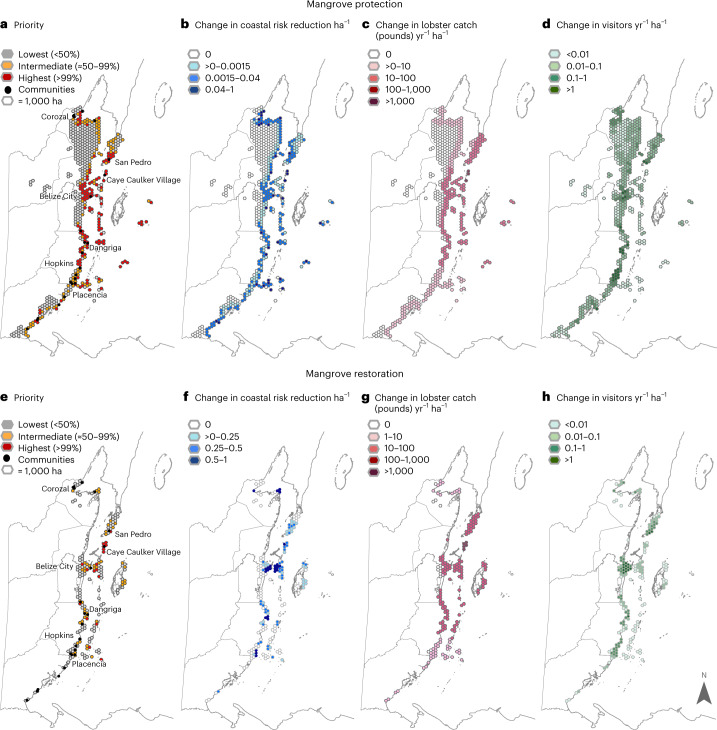
Priority locations and co-benefits of selected blue carbon targets. **a**–**d**, Priority locations (**a**) for investing in the submitted NDC target of 12,000 ha mangrove protection with corresponding changes per ha of mangrove protected in coastal risk reduction (**b**), lobster fishery (**c**) and tourism benefits (**d**). **e**–**h**, Priority locations (**e**) for investing in submitted NDC target of 4,000 ha of mangrove restoration with corresponding changes per ha of mangrove restored in coastal risk reduction (**f**), lobster fishery (**g**) and tourism benefits (**h**). Priority is based on the number of times a hexagon is selected out of 1,000 model runs. Results for coastal risk reduction are rescaled from 0 to 1. Note that the results are per unit hectare of mangrove and not per hexagon.

By optimizing ecosystem services for the proposed protection and restoration targets, we show where to prioritize investments in the nature-based solutions needed to achieve a portfolio of adaptation co-benefits (Fig. 3a,e in red and orange). A key component of the optimization analysis involves quantifying where improved delivery of co-benefits would be greatest for the least area of investment with implementation of the proposed targets. We did not incorporate costs as an objective, but including the area of the restoration and protection targets as a constraint in the optimization (Methods) serves as a proxy for cost and accounts for per unit area service production. For mangrove protection, we find the greatest benefits of risk reduction in northern Belize near Corozal, around Belize City, and in the southernmost part of the country near Punta Gorda town. These patterns are due to spatial variation in waves, wind, shoreline type and the extent of other ecosystems that provide coastal protection benefits (for example, seagrass, coral) (Fig. 3b). We find the greatest tourism benefits near San Pedro, Dangriga, Hopkins and Placencia, all areas with relatively high visitation currently and intact mangrove systems under threat from development (Fig. 3d). Change in lobster benefits is greatest in north central and central Belize where catches tend to be higher (Fig. 3c). In areas such as Caye Caulker, where coastal vegetation has been lost to development, our results suggest the remaining intact mangroves are highly important nursery habitat for young lobster before they migrate offshore. We find somewhat contrasting results for restoration. The highest priority areas are concentrated around population centres (for example, Belize City, San Pedro, Cay Caulker, Dangriga, Hopkins) in places where mangroves are currently degraded but have the potential to reduce risk, support tourism and provide nursery habitat to key fishing grounds (Fig. 3e–h). These results demonstrate the importance of assessing ecosystem services provided by blue carbon strategies using models that account for the physical and social factors that influence a suite of co-benefits and not just assume that anywhere mangroves are protected or restored will provide equal benefits^25^.

**Fig. 4:**
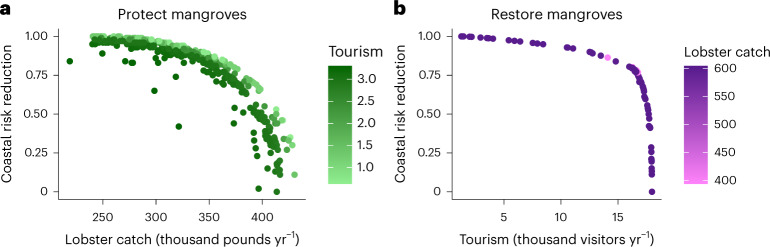
Efficiency frontiers for co-benefits of selected blue carbon targets. **a**,**b**, Efficiency frontiers showing optimal solutions between lobster catch (thousands of pounds per year) and coastal risk reduction (hazard index normalized to 0–1) on the *x* and *y* axes and tourism (in thousands of visitors per year) in the green scale for the protection target (**a**) and tourism and coastal risk reduction on the *x* and *y* axes and lobster catch in the purple scale for the restoration target (**b**).

**Extended Data Fig. 3:**
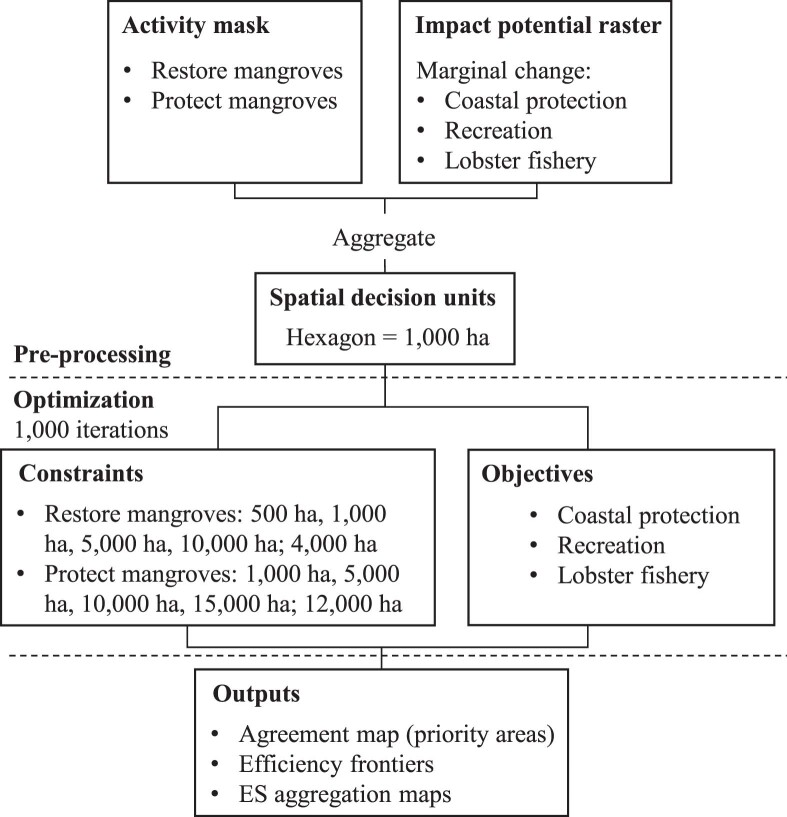
Overview of the ROOT optimization workflow. The workflow includes data inputs, the constraints (which represent the targets) and objectives (co-benefits) for the optimization, and the outputs.

**Extended Data Fig. 4:**
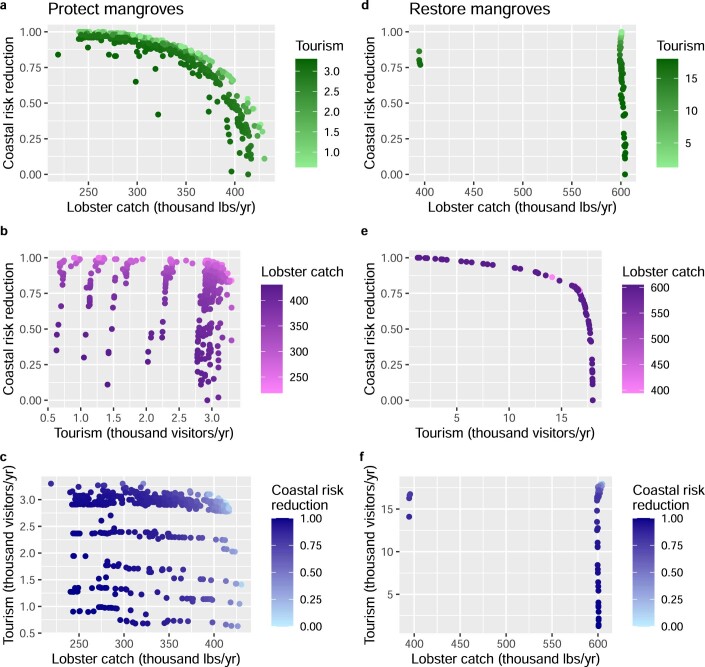
Efficiency frontiers for co-benefits of selected blue carbon targets. Efficiency frontiers for the 12,000 ha mangrove protection target (**a–c**) in the left column and 4,000 ha mangrove restoration target (**d–f**) in the right column. These plot a set of possible solutions from the optimization analysis that prioritize the three co-benefits. We show the two-way relationships with the third ecosystem service in the colour scale for each combination of co-benefits. The units of the ecosystem services in the colour scale are the same as the units on the x and y axes. The lines in panels b, c, d, and e display when sub-optimal solutions in our analysis occur, such as when the solutions for mangrove protection leave out top tourism locations (for example, Ambergris Caye and Placencia).

Our results also highlight the potential pitfalls of single-objective planning. For example, if investments in mangrove protection were made based solely on lobster, our analysis suggests prioritizing the shoreline south of Belize City, which with more protection from the barrier reef and less infrastructure is a lower priority for coastal risk or tourism benefits. By conducting a multi-objective optimization analysis using the management targets as spatial constraints (Methods and Extended Data Fig. 3), we derive a set of efficiency frontiers that maximize net benefits across a range of preferences for coastal risk reduction, fisheries and tourism (Fig. 4 and Extended Data Fig. 4). The priority locations for implementation (Fig. 3a,e) maximize bundles of adaptation co-benefits in addition to carbon storage and sequestration, thus offering a more efficient pathway to climate resilience relative to considering nature-based solutions for each sector separately. Furthermore, our analyses suggest that implementation of the NDC targets adopted by the Belizean government will lead to spatial management of mangroves that produce a suite of ecosystem service benefits along the edge of the efficiency frontier rather than producing sub-optimal solutions^37^. Such application of efficiency frontiers is well established in the ecological–economics literature on marine spatial planning^37,38^ and landscape management^39,40^. We advance this space by demonstrating the power of multi-objective optimization for informing quantitative targets for nature-based solutions in NDCs.

## Discussion

By spatially quantifying multiple benefits of blue carbon strategies, we reveal where more ambitious targets for mangrove protection and restoration generate greater carbon storage and sequestration, tourism, fisheries and coastal risk-reduction benefits. Our results also provide scientific information about where to direct efforts and investment to achieve a suite of climate mitigation and adaptation goals, thus allowing Belize to operationalize blue carbon solutions in its NDC more efficiently. The diminishing returns in co-benefits from greater hectares of protected and restored mangroves and the priority areas for investment offer Belize—and other countries seeking to increase the ambition and efficiency of their NDCs—a hopeful pathway for balancing coastal development with ecosystem protection to foster a sustainable ocean-based economy.

Blue carbon strategies are an opportunity to promote synergies between climate action and multi-sectoral economic development^12,34,41^. Belize’s first NDC, submitted in 2016, consisted of mitigation objectives for multiple sectors including forestry, electricity, waste and transport and a description of near-term adaptation actions. The mangrove protection and restoration objectives lacked specific targets but did anticipate avoiding up to 379 Gg of CO_2_ emissions between 2015 and 2030^42^. That same year, the Integrated Coastal Zone Management Plan helped to strengthen frameworks for conserving key ecosystems that underpin the main drivers of the economy and social resilience: tourism, fisheries and reducing costs from disasters such as coastal storms^25,32^. Now the updated NDC builds on and advances the Integrated Coastal Zone Management Plan by quantifying the benefits of nature-based solutions and highlighting the potential interplay among multiple sectors for climate action and finance^4^. Demonstrating how investments in ecosystems support long-term economic growth is key for centering green infrastructure projects in national development and climate resilience programmes where there may be greater resources than environmental policies and ministries^12,31,34^.

Our iterative approach of evaluating blue carbon targets by quantifying their multiple benefits in terms of ecosystem services can also inform monitoring and verification of nature-based climate commitments. Traditionally, restoration monitoring has focused on ecological attributes such as area or density of vegetation at local scales. As an increasing diversity of actors invest in green infrastructure, there is growing interest and need to track social and economic outcomes from nature-based interventions at larger spatial scales^43–46^. Ecosystem service models provide metrics that link biophysical changes in system structure and function with societal values that resonate with local beneficiaries and investors.

There are, of course, limitations to spatial prioritization as a decision-making tool^47^. Wyborn and Evans^47^ call attention to prioritization studies that suggest, often problematically, that a global analysis can be readily used to inform local actions. They also highlight the ways in which modelling exercises often bias quantitative information and render qualitative data less relevant. In Belize, we integrated the prioritization into a national policy process with ongoing involvement in the data collection, framing, modelling and interpretation by local experts (many of whom are authors on this paper). Co-creation of the analysis in the context of a broader planning effort is likely to increase the impact of the prioritization and dampen the risk it will crowd out other social and ecological objectives and ignore diverse contexts and ways of knowing^2,25,34,47^.

Belize’s updated NDC provides a triple win. By committing to—and fully implementing—protection of an additional 12,000 ha of mangrove forest by 2030, the country safeguards double the area of mangroves contributing to the national carbon sink in the future. In combination with restoration commitments, these mangroves play a critical role in achieving Belize’s low emission development strategy, which aims to reduce national emissions by 86% up to 2050. They also buffer impacts to coastal infrastructure and provide for the safety of its citizens. By expanding and protecting habitat for ecologically and economically important species, blue carbon ecosystems support livelihoods through tourism and fisheries.

Belize’s science-driven approach to NDC design, built on ex ante measurements of the impact of coastal wetland conservation and restoration in terms of greenhouse gas (GHG) benefits and community, economic and environmental co-benefits, has helped formulate specific and verifiable targets, while also informing implementation with location-specific details. However, achieving these outcomes will depend on more than just area-based targets for mangrove restoration and conservation. Mangroves are also influenced by the condition of inland and ocean ecosystems^48^. Watershed management, coral reef and seagrass protection and other integrated strategies are necessary for mangrove health and persistence. Belize’s updated NDC acknowledges the need for integrated ridge-to-reef management, including targets related to revising and streamlining current coastal zone legislation to close existing gaps^13^.

The updated NDC also considers the importance of biodiversity for long-term resilience and climate mitigation and adaptation. For example, tourists are drawn to the diversity of birds, fish and invertebrates that mangroves support, many of which generate important subsistence and commercial fishery benefits. Effective coastal risk reduction also relies on diverse coastal areas, with coral, mangroves and seagrasses together providing multiple lines of defence^49^. While we did not explicitly include biodiversity as an objective in the optimization analysis, biodiversity both underpins ecosystem services^50^ provided by mangroves and benefits from implementation of blue carbon targets.

Questions about how on-the-ground interventions and projects lead to changes in mangrove structure and function and in turn changes in ecosystem service benefits over time deserve further attention^17,51^. For example, in reality, neither protection nor restoration will be 100% effective; nor would the baseline situation (if neither strategy were implemented) likely lead to full habitat loss. Moreover, the current study assumes restored mangroves are functionally equal to protected mangroves in terms of their ability to provide co-benefits, when in reality, the vegetation may not provide fully functional nursery habitat, coastal risk reduction and tourism opportunities by 2030^51,52^. In addition, sea-level rise and other climate-related changes will probably influence habitat suitability for mangroves and the carbon and co-benefits they generate. If Belize is able to fill key information gaps, such as comprehensive light detection and ranging (LiDAR) data, future NDC processes could model climate scenarios to explore how highest priority areas for protection or restoration may change over time with rising seas and warming temperatures.

Growing research and technology in Earth observations will also improve the ecological data (for example, trunk density, canopy height) needed to spatially model the social and economic benefits of ecosystem services and to monitor and verify outcomes, including time lags in service provision. Empirical observations of total ecosystem carbon stocks are underway in Belize and will address limitations of this study in future NDC updates. By drawing in part on data from the Caribbean shoreline of Mexico, which has less riverine, lagoonal and island mangrove ecosystems than Belize, our study may conservatively estimate carbon stock (Methods). Although the models used in this study differ in major sources of uncertainty, previous research demonstrates confidence in their results via comparison to observed data (Methods). Our use of area as a proxy for restoration and protection costs assumes the resources needed to implement these strategies do not vary from location to location. Further work at local scales could inform a full cost–benefit analysis for mangrove restoration and protection efforts that would require spatial data on costs, property values and land tenure. In the future, comprehensive carbon accounting could incorporate the additional carbon footprint from increases in tourists and fisheries.

The global picture is dire. Rising temperatures since the Paris Accord mean countries need to increase their ambition for climate response at a faster pace to keep up with the shifting realities^53,54^. Developing countries need financial, scientific and capacity-building support to increase their level of ambition for reducing emissions and building resilience to the effects of climate change^53^. The pioneering example of ambition that Belize sets here can be emulated by other countries aiming to strengthen their NDCs to meet agreements in the Glasgow Climate Pact signed at COP26. Our study lays out a general approach for quantifying co-benefits of nature-based solutions in terms of ecosystem services that support climate adaptation and carbon storage and sequestration.

Strategically designed nature-based approaches can help in meeting more ambitious climate goals and address the needs of multiple sectors (for example, fisheries, disaster risk reduction, tourism). Belize has demonstrated the benefits of a cross-sectoral, multi-stakeholder science–policy process for designing and committing to more ambitious nature-based climate mitigation and adaptation targets. Ongoing support for implementation through policies and practices that can bolster protection and capacities to carry out restoration and track outcomes is needed around the world to meet GHG mitigation commitments and design climate-resilient societies^27^.

## Methods

We developed an approach for evidence-based target setting of nature-based solutions for a country’s NDC and applied it in Belize. The workflow consists of two main parts: (1) evaluating possible blue carbon targets and (2) estimating benefits and prioritizing implementation of selected targets. Below we describe the methods for each of the steps in the workflow including designing and mapping the two blue carbon strategies, quantifying carbon storage and sequestration, quantifying co-benefits, optimizing co-benefits and comparing priority areas to key data layers to support target selection. We repeat several of these steps in the two main parts of the workflow as shown in Extended Data Fig. 1. The workflow is meant to be iterative to highlight the adaptive nature of the NDC process under the Paris Climate Agreement. During this process, we engaged representatives from the Belizean national government, local and international environmental non-governmental organizations (NGOs) and academia through engagement with the Blue Carbon Working Group.

### Engagement with Blue Carbon Working Group

Our main approach to eliciting broader input into the blue carbon targets for the update to Belize’s NDCs was to leverage engagement with the Blue Carbon Working Group. The working group was established by World Wildlife Fund and the government of Belize in 2020 to help provide technical inputs and guidance on efforts to enhance protection of Belize’s coastal ecosystems through updates to the country’s NDCs and other policies, plans, strategies and regulatory instruments. The working group consisted of 14 members representing a diversity of government, NGO and academic institutions. The membership included seven representatives from government agencies (that is, Belize National Climate Change Office, Belize Coastal Zone Management Authority and Institute, Belize Forest Department, Belize Fisheries Department, Belize Ministry of Natural Resources, Belize National Spatial Data Infrastructure, Belize National Biodiversity Office). Membership also included three representatives from environmental NGOs (that is, World Wildlife Fund, MARFUND, Association of Protected Areas Management Organization), three academic institutions (that is, University of Belize Environmental Research Institute, Stanford University, University of Alabama Huntsville) and one foundation (that is, Pew Charitable Trust).

The Working Group met quarterly from early 2020 through 2021 to make progress on a variety of efforts related to integrating blue carbon ecosystems in the updated NDC. During three of these meetings (August 2020, October 2020, February 2021), we included agenda items related to the optimization analysis documented in this study. During the first meeting, we introduced the inputs, outputs and scientific underpinnings of the models for quantifying ecosystem services. We also asked working group members to explain the specific actions (for example, plantings, hydrological interventions, enforcement of existing protection laws, designation of new protected areas) involved in protecting and restoring mangroves in Belize and used this information help guide our design of the blue carbon strategies (Design and map blue carbon strategies section below). During the second exchange with working group members, we shared initial results for a set of possible targets for the area of mangrove protected (that is, 500; 1,000; 5,000; 10,000; these were earlier versions of the priority area maps shown in Fig. 2). We asked members if these options sufficiently captured the potential area-based targets they wanted us to explore. On the basis of their feedback that they wanted to explore a larger target area, we revised the set of protection targets to those reported in this study (5,000; 10,000; 25,000), added restoration targets (Fig. 2) and re-ran the analysis. In February 2021, we shared optimization results for the selected targets with the Blue Carbon Working Group and discussed implications for the implementation of the blue carbon targets within the NDCs.

In addition to gathering information to design the protection and restoration strategies and select potential targets to explore, we leveraged the Blue Carbon Working Group meetings for three other objectives. These included (1) understanding their goals for incorporating blue carbon strategies in the NDCs, (2) eliciting feedback on the input data we used in the blue carbon NDC analysis and (3) providing an opportunity for government entities, environmental NGOs and local scientists to evaluate findings and guide next steps. We accomplished these objectives through participating in working group discussions, sharing slide decks with maps of the results (especially related to versions of Figs. 2 and 3), asking the participants how the patterns in the results did or did not resonate with their local experience and knowledge and discussing options for revising the analysis to incorporate their expertise. All of the activities and outcomes of the meetings, including the aim to publish this academic article, were agreed upon by the group of participants. None of the working group members were individually affected by the analyses conducted in this study.

### Design and map blue carbon strategies

We designed two blue carbon strategies—mangrove protection and restoration—based on engagement with the Blue Carbon Working Group (above), outputs from a previous project on climate adaptation across the Mesoamerican Region (that is, including Mexico, Belize, Guatemala and Honduras) and historical data on mangrove health and degradation. A key outcome of the previous project was a list of ecosystem-based adaptation strategies of interest to each country. For Belize, two of these strategies were mangrove protection and restoration. Because these strategies are also blue carbon strategies, we selected them for inclusion in the NDC analysis.

We specified mangrove protection as the maintenance of intact habitat through enforcement of existing protection laws and designation of new protected areas. Protection also includes strengthening regulations related to clearing mangroves on private land and considering land trusts and incentives related to blue carbon. This strategy can occur within or outside protected areas and on public or private land in places where mangroves are relatively healthy. To identify these locations, we used a spatially explicit risk assessment that maps loss and fragmentation of Belize’s mangroves based on historical disturbance over a 30-year time series^55^. Using a metric related to patch irregularity, the study categorized ‘fragmentation risk’ into six classes from ‘very high: frequently fragmented’ to ‘very low: not fragmented before’. On the basis of conversation with Belizean partners and experts (Engagement with Blue Carbon Working Group section), we used the metric for fragmentation risk to identify healthy vs degraded mangrove. We considered mangroves with no historical fragmentation (that is, only the ‘very low’ class as ‘healthy’ and thus appropriate for protection. The total area of healthy mangroves is the full opportunity area for implementing the mangrove protection strategy (Extended Data Fig. 2a).

We specified the mangrove restoration strategy as the revitalization of degraded or cleared mangroves through plantings and hydrological interventions. To identify opportunity areas to implement these actions, we considered areas where mangroves had been cleared in the past 39 years (1980–2019) and existing mangroves with a history of fragmentation. Cleared areas are based on a time series of mangrove extent from 1980–2019^36^. Degraded areas are based on the risk assessment described above and used to identify healthy mangroves^55^. We considered any mangroves that had seen some degree of fragmentation in the past (five of the six risk classes mapped in the aforementioned analysis) to be either degraded or subject to degradation and thus be a candidate for restoration. The total area of cleared mangroves and mangroves at risk of degradation is the full opportunity area for mangrove restoration (Extended Data Fig. 2b).

### Quantify carbon storage and sequestration

To quantify total carbon stock provided by Belizean mangroves, we estimated storage and sequestration for the protection strategy and sequestration for the restoration strategy. For protection, we included the standing stock that would be lost if the healthy mangroves were converted to another land use and the accumulation of carbon that would have occurred over a set period (in this case, 30 years). For the restoration scenario, we estimated sequestration because it takes decades for the carbon stocks to accumulate in the trees and soil. We used published carbon stock data from the Mexican part of the Yucatán Peninsula that varies with tree stature^56^, IPCC Tier 1 values for growth rate and root-to-shoot parameters^15^ and spatial data on mangrove extent and stature from Belize^36^. At the time of Belize’s update of its NDCs and the writing of this paper, countrywide carbon stock data for mangroves collected in September 2021 were still being analysed. These surveys were delayed due to the COVID-19 pandemic. Thus, country-specific values for Belize could not be used in the current analysis nor in the 2021 update to Belize’s NDC.

For the protection strategy, we estimated the area occupied by dwarf, medium and tall mangroves and mangrove savannas (which we characterized as medium stature) within the selected 12,000 ha target and calculated the proportion of area that each tree stature occupied. We assumed mangrove carbon stocks (above- and belowground biomass) for each tree height (dwarf, medium and tall) based on estimates from Quintana Roo, Mexico^56^ (the coastal region just north of Belize) and included dead organic matter stocks where applicable (medium and tall stands but not dwarf stands^56^). We calculated soil carbon stocks to 0.5 and 1 m to capture variation in potential oxidation with drainage^56^. We report the results for 1 m in the manuscript and include the 0.5 results in the supplement for reference (Supplementary Table 3). For the biomass accumulation rate, we used the IPCC default aboveground growth rate for dry tropical mangroves (3.3 t ha^−1^) and the IPCC default value for subtropical root-to-shoot ratio (0.96) to estimate belowground biomass growth^15^ and converted biomass to carbon using 0.48 and 0.39 for above- and belowground biomass, respectively^57,58^. The soil carbon accumulation rate from VM0033 (1.46 t C ha^−1^ yr^−1^) (ref. ^59^) was used, which is more conservative than the IPCC default value of 1.62 t C ha^−1^ yr^−1^.

The yearly removals were calculated by multiplying the area of each mangrove stature by the biomass and soil carbon sequestration rates over 30 years. Thirty years is the amount of time assumed for a tree to reach maturity under the restoration strategy. For consistency, we applied this same time period to the protection strategy because even in mature forests, there is tree growth, accumulation of litter, dead standing wood and soil carbon accumulation that would be lost if a mangrove stand were developed. We calculated the standing stock by multiplying the area of each mangrove type by the total tree carbon stock and dead organic matter (when applicable). These values were summed for the total removals over 30 years. While both methane and nitrous oxide can be produced in mangrove ecosystems and reduce the amount of carbon ultimately sequestered^60^, these GHGs were not included in this analysis due to lack of appropriate emissions data and spatial data on where production could occur. To estimate carbon storage and sequestration for the range of protection targets, we used the same ratios of mangrove stature from the 12,000 ha target selected by the Belizean government, multiplied those by each target area (5,000, 10,000, 20,000, 25,000 and 64,000 ha) and followed the same methods as above.

For the restoration scenarios, we followed a similar approach. Within the area of the 4,000 ha restoration option, we calculated the proportional area of each mangrove stature^36^. We calculated above- and belowground tree and soil carbon accumulation as described above. Because the literature lacks known growth rates for each mangrove height in this climate zone, we applied the same values across dwarf, medium and tall mangroves and mangrove savannah. We assumed that carbon accumulation began one year after restoration and that the total carbon removals were based on 30 years of restoration. To scale to the different restoration target areas (500, 1,000, 5,000 and 13,000 ha), we used the same ratios of mangrove stature from the 4,000 ha target and multiplied those by each target area and followed the same methods as above.

The main limitation of the carbon component of this study is the lack of stock data for Belize. Total ecosystem stocks vary with sediment availability and geomorphic setting (for example, riverine system versus island) and thus may be quite different depending on location^61^. Due to the proximity and similar geomorphic setting of the Mexico study^56^, it is reasonable to assume that the relationship between carbon stock and tree stature for Mexico is applicable to Belize. That said, Belize contains a mix of riverine, lagoonal and oceanic island mangrove ecosystems, which are not all captured in the Mexico dataset^56^. Because the total carbon stock of these ecosystems tends to be higher than that of mainland ecosystems (especially the soil stock), the values applied in this study are probably a conservative estimate. Note that despite the lack of country-specific carbon stock data, the analysis still contains spatial variation in carbon stock due to the Belize specific spatial data for tree stature and the Mexico stock data by stature. Restoring wetlands has the high potential to capture and store carbon, but the actual outcome and benefits will strongly depend on how restoration actions influence the ecology of mangrove systems and how they are managed over time^51,52^.

### Quantification of co-benefits

To evaluate the multiple benefits of blue carbon strategies and priority locations for their implementation, we quantified the influence of mangrove restoration and protection on three ecosystem service co-benefits: coastal risk reduction, spiny lobster catch and revenue and visitation and expenditure from tourism. We used a suite of ecosystem service models (below) to estimate the expected change in benefits provided by mangroves under each strategy. We calculated expected marginal change in each ecosystem service (equation (1) at a 30 m grid-cell resolution.

1 $${{V_\mathrm{marginal}}}=|{{{V}}}_{{\rm{strategy}}}{{-}{{V}}}_{{\rm{baseline}}}|$$

In the equation above, *V*_marginal_ represents the marginal change in benefit (or ecosystem service) with implementation of the strategy, *V*_strategy_ is the magnitude of the ecosystem service under the mangrove restoration or protection strategy for the possible or selected targets and *V*_baseline_ is the magnitude of the ecosystem service under the present extent of healthy mangroves (using 2019 mangrove data). For the mangrove baseline layer, we assumed that degraded or cleared mangroves were not capable of providing ecosystem services. The mangrove layer for the restoration strategy included the full opportunity area for restoration and the existing healthy mangroves. We assumed the existing healthy mangroves would stay healthy and degraded mangroves would become healthy with implementation of the restoration targets. Thus, the mangrove footprint to assess the influence of restoration was larger than the baseline area. To quantify the marginal value of ecosystem services under the protect mangroves strategy, we compared the baseline to the potential loss of all currently healthy mangroves if these were not protected and without additional restoration. We then used the spatially explicit differences in ecosystem services for the full opportunity area of the two blue carbon strategies as inputs into the optimization analysis. We also quantified co-benefits of the selected targets using the same approach and equation (1). Lastly, for both restoration and protection, we made the simplifying assumption that the strategy would be fully implemented and that previously degraded mangroves would be capable of providing ecosystem service co-benefits (service specific methods below) by 2030. While Belize is actively selecting areas to implement the targets, full restoration of some particularly degraded mangroves may be unlikely by 2030. It will be important to account for potential time lags in the delivery of ecosystem services when developing a monitoring and evaluation plan.

### Optimization of ecosystem services

To explore a range of possible targets (Supplementary Table 2) and identify priority locations for mangrove protection and restoration, we conducted an optimization analysis of three ecosystem services. We used the Restoration Opportunities Optimization Tool (ROOT), an open source model available at naturalcapitalproject.stanford.edu/software/root ref. ^62^. Employing a multi-objective spatial optimization approach^39^, ROOT is a software tool that provides decisionmakers with information about how to optimize trade-offs among multiple spatially explicit ecosystem services associated with different management strategies (for example, blue carbon strategies for climate mitigation and adaptation). ROOT requires four main inputs for each strategy: (1) activity mask raster, (2) impact potential rasters, (3) constraints and (4) objectives (Extended Data Fig. 3).

The activity masks identify at a grid-cell level the areas where strategies can occur (Designing mangrove strategies section above). In our analysis, the activity masks are maps of the full opportunity area where mangrove protection and restoration could be implemented (Extended Data Fig. 2). These are the locations considered in the optimization. The impact potential rasters are maps of the expected marginal change in the biophysical supply of an ecosystem service at the grid-cell level if a strategy is implemented (Quantification of co-benefits section above). The constraints correspond to resource constraints (for example, budget) or policy goals (for example, NDC targets) that can limit the total area available to implement the strategy. In our analysis, we set constraints that specified the amount of area for implementing mangrove protection and restoration. First, we explored a range of possible constraints/targets (Supplementary Table 2 and right side of Extended Data Fig. 1). Next, we conducted an optimization analysis using the selected NDC targets as spatial constraints: 4,000 ha and 12,000 ha for restore and protect, respectively. We set the management objectives as the ecosystem service co-benefits: coastal risk reduction, tourism and lobster catch.

ROOT uses spatial decision units (SDUs) to identify specific areas to which actions are allocated. We employed a hexagon grid of 1,000 ha size automatically generated by the ROOT user interface as our decision units. It aggregates values from all data inputs by these decision units and considers only the units that fall within the activity mask raster, which for this analysis resulted in a total of 1,196 and 1,678 decision units for restoring and protecting mangroves, respectively. We then calculated for each SDU the total value of ecosystem services provided if the given strategy is implemented in each potential pixel (30 m resolution; Quantification of co-benefits section). Using ROOT, we carried out a number of independent linear optimizations (1,000 iterations) that randomly assign weights to each objective (or ecosystem service) to identify locations that maximize benefits per unit area of intervention (protection or restoration). The optimizations are formulated with a weighted-sum objective function:

$$\mathop{{\rm{max }}}\limits_{K}\sum _{i}\sum _{s}{w}_{s}{v}_{{si}}({k}_{i})$$

where *K* represents possible decisions across all SDUs, *w*_*s*_ is the weight assigned to objective (or ecosystem service) *s* in this iteration of the optimization and *v*_*si*_(*k*_*i*_) is the value to objective *s* given that *i* is assigned activity *k*_*i*_. Each optimization was conducted subject to the constraints specified above. Varying the weights across the range of all possible weight combinations in this manner allows decisionmakers to visualize the trade-offs associated with a full range of preferences for different ecosystem services (Fig. 4 and Extended Data Fig. 4)^39,62^.

We produced three main sets of results from the ROOT outputs (Extended Data Fig. 3): (1) the ‘agreement maps’ that show the highest priority locations for investing in the mangrove restoration and protection strategies (Figs. 2 and 3a,e), (2) the efficiency frontiers that illustrate three-way trade-offs (in two-dimensional space) between the services generated for each possible spatial configuration of the strategy that would meet the target area of implementation (Fig. 4 and Extended Data Fig. 4) and (3) the aggregated ecosystem service maps that show the change in each ecosystem service if the strategies were implemented in the highest priority locations (Fig. 3b–d,f–h). We identified the highest priority areas by classifying the SDUs that were selected more than ≈50% of the time (orange) and more than 99% of the time (red) by the optimization analysis. Thus, the highest priority areas, as shown in the agreement maps, are a synthesis of the hexagons frequently selected by the optimization analysis (when the relative weighting of objectives is varied), rather than a single optimal spatial solution along the frontier. Together the orange and red areas in the priority maps for the final targets (Fig. 3a,e) meet the proposed 12,000 ha of mangrove restoration and 4,000 ha of mangrove restoration, respectively.

### Comparison of priority areas to existing legislation, MPAs and focal communities

We spatially compared the priority areas for the range of potential targets to several key pieces of information to inform the target selection process. We overlaid the priority areas with maps of mangroves selected for protection in existing mangrove legislation^63^. This comparison identifies locations selected both by the models—as highest priority sites for implementing mangrove protection because they would generate the greatest delivery of multiple ecosystem services—and as identified through the political process (Fig. 2). We also overlaid the priority area maps with the location of existing marine protected areas (MPAs). This comparison shows which priority areas for mangrove protection as identified by the models already have some protected status (Fig. 2). Finally, we overlaid the priority areas for restoration with maps of local communities that could benefit from ecosystem services. This comparison highlights where mangrove restoration is most likely to deliver multiple ecosystem services to particular populations. These spatial comparisons were used in discussions with stakeholders and government officials during the target selection process.

### Ecosystem service modelling

#### Coastal risk reduction

To quantify coastal risk reduction provided by mangroves in Belize, we calculated a hazard index for coastal erosion and flooding using the InVEST Coastal Vulnerability model^64^. The InVEST software suite consists of multiple ecosystem services models and is open source and available for download at https://naturalcapitalproject.stanford.edu/software/invest. The InVEST Coastal Vulnerability model advances previous similar coastal hazard indices^65–67^ by explicitly considering the role of ecosystems, such as mangroves, in providing coastal protection and incorporating information about people, property and other relevant metrics in the framing of risk^19,64,68–71^.

The index quantifies the relative exposure of a given stretch of shoreline to flooding and erosion based on the following variables: the diversity and extent of coastal and marine ecosystems, coastal elevation, exposure to waves and wind, shoreline geomorphology, storm surge potential and sea-level rise. For each segment of coastline in a given area of interest, the data for the seven variables are assigned ranks from lowest exposure (rank = 1), to highest exposure (rank = 5) based on a combination of absolute and relative modelled and observed data (Supplementary Table 4). The final coastal hazard index is the geometric mean of the ranked variables (where *R* = rank and all variables are given equal weighting).

$$\mathrm{HazardIndex}={\left({R}_{\mathrm{Habitats}}{R}_{\mathrm{ShorelineType}}{R}_{\mathrm{Relief}}{R}_{\mathrm{Waves}}{R}_{\mathrm{Wind}}{R}_{\mathrm{SurgePotential}}{R}_{\mathrm{SLR}}\right)}^{1/7}$$

For the country of Belize, we assessed exposure to coastal hazards and risk reduction provided by mangroves along the entire coastline of the mainland and cayes at a 250 m resolution. On the basis of prior research and engagement with stakeholders, policymakers and scientists, we identified coastal and marine ecosystems that provide critical risk-reduction benefits for Belizeans, including corals (barrier and fringing reef), mangrove forests, coastal forests (non-mangrove) and seagrass beds^25,49^. We then used the best available data on habitat extent to create comprehensive spatial footprints of each of the four types of habitat (Supplementary Table 1). Each habitat type was assigned a rank based on differences in morphology and expected ability to prevent erosion and attenuate waves and storm surge (Supplementary Table 4; refs. ^19,67–70^ provide more information and previous applications of this approach). The habitat ranks were informed by expert judgement and the peer-reviewed literature^19,20,72–74^. A rank of ‘1’ offers the greatest protection, ‘4’ the least and ‘5’ designates no protection afforded by habitat. The coastal hazard index does not include mathematical formulations representing wave or surge attenuation due to frictional effects or drag effects of mangroves on water flow (for example, ref. ^73^). A habitat-specific ‘protective distance’ was also defined to indicate the extent of coastline probably receiving protection from a given habitat type (Supplementary Table 5). These distances are essentially a technical shortcut, rather than an ecological or hydrodynamic parameter. They allow us to designate which coastline segments are protected by patches of habitats located at different distances from the grid cells, given that the model does not take into account the numerous factors (depth, channel configuration, distance from the coast and so on) that could influence the distance over which effects of these habitats may be prominent^19,64,70^.

For waves, wind, relief and storm surge potential, we used globally available datasets, including the National Oceanic and Atmospheric Administration’s Wave Watch 3 model hindcast that catalogues wind and wave statistics over multiple years, the SRTM 30 m global topography layer used to calculate average coastal elevation and a global map of the continental shelf margins used to compute a proxy value for storm surge potential, which is ranked based on the distance from the shoreline to the edge of the shelf. Shoreline type is a national scale dataset created using satellite imagery by the team. Supplementary Table 1 provides a detailed list of data sources, and refs. ^19,70^ provide further details related to data processing. As there is little documented spatial variation in the rate of sea-level rise across the geographically small study area, we did not include sea-level rise as a variable in the analysis. This does not mean that sea-level rise does not influence risk to coastal erosion and flooding. It simply means that coastal risk reduction provided by mangroves would not differ substantially across the study area because of variation in sea-level rise.

The results from the coastal hazard index demonstrate where mangrove risk-reduction benefits are delivered (that is, the coastline), not where the ecosystem service is provided (that is, by which sections of mangrove forest). Because ROOT is designed to evaluate where on the land or seascape to target blue carbon management actions for the greatest returns, we developed an approach to backtrack the coastal risk-reduction benefits delivered at the shoreline to the grid cells of mangrove providing the service. For each 30 × 30 m grid cell of mangrove, we calculated an average ‘impact value’ using the 2 km protective distance associated with mangrove habitat (Supplementary Table 5) as the search radius around each pixel that identified the shoreline segments to which that pixel of mangroves was helping to reduce risk. We then standardized the impact value for each pixel by the area of mangroves (ha) in each ROOT spatial decision unit (SDU = 1,000 ha) providing coastal protection (that is, each unit within 2 km of the shoreline). While more extensive habitat probably provides more coastal protection, the relationship is not linear (that is, at some point wave attenuation levels off and additional habitat area does not provide additional coastal protection benefit^72,75^). This approach allowed us to estimate the marginal difference in coastal risk reduction with implementation of the two mangrove strategies compared to the baseline (Fig. 3b,f). We then used these results as inputs into the optimization analysis (above).

The model limitations and assumptions are described at length in refs. ^19,70^. Briefly, the index is most appropriate for understanding relative differences in risk reduction provided by ecosystems along the shoreline and requires assumptions about how far inland exposure to hazards will propagate. Furthermore, the index uses a proxy for surge potential that may oversimplify storm dynamics that are complex and can result in unexpected scenarios such as the negative surge associated with hurricane Irma^76^. The habitat ranks represent differences in the relative ability of different nearshore and coastal ecosystems to attenuate water flow. These are based on literature review and ultimately lack information about specific mechanisms. Nevertheless, despite these limitations, several studies have found good correspondence between areas of high risk, as estimated by the InVEST Coastal Vulnerability model and empirical data on impacts from coastal hazards^19,68^.

#### Spiny lobster catch and revenue

To quantify benefits of mangroves for fisheries, we modelled the catch of Caribbean spiny lobster (*Panulirus argus*) using an age-structured population model with Beverton–Holt recruitment that was developed for previous work in Belize and described in length elsewhere^25,77^. In brief, we modelled the Belize lobster population as nine regional, linked, subpopulations each associated with one of nine subregions (subregions correspond to nine coastal planning regions as delineated in the Belize Integrated Coastal Zone Management Plan^25,32,35^). These subregions are connected via immigration as lobster move from mangroves and seagrass (nursery habitats) in larval and juvenile life stages to seagrass and coral reefs as they enter adult life stages; survival is dependent on availability of juvenile and adult habitat. Initial conditions are based on the area of mangrove and seagrass (habitat for larvae and juveniles) and coral reef and seagrass (habitat for adults) in each planning region. In this analysis, we assessed the impact of mangrove protection and restoration activities on the catch of spiny lobster to inform the prioritization of these strategies. For restoration, we assumed that all restored mangrove became healthy and were capable of providing juvenile and adult habitat for lobster. For protection, we quantified the marginal value of the healthy mangroves by calculating the change in lobster catch if all healthy mangroves were degraded and no longer able to provide habitat for lobster (Quantification of co-benefits section). All the model equations and parameter values are the same as those used in previous applications of the model^25,77^, with the exception of the price per pound of lobster tail meat, which we updated to the 2019 (pre-COVID) market rate of $26.17 BZD (https://www.selinawamucii.com/insights/prices/belize/lobster/).

We calculated baseline catch using the current extent of healthy seagrass, coral reef and mangrove habitat in the nine planning regions, incorporating catch data from the Belize fisheries department^78^ and life history parameters from regional literature^79–83^. Only mangrove within 250 m of the shoreline was considered as nursery habitat. Changes in lobster catch associated with protection or restoration of mangrove habitat were modelled at the subregion scale, and the impact potential of the mangrove strategies was the marginal change in catch for the whole Belizean fishery associated with a change in mangrove extent in a subregion. Because lobster migrate between subregions, a change in the habitat available in one subregion may influence the catch produced by other subregions for which it functions as a source. To create inputs for ROOT, we converted the subregion marginal value in catch to a per pixel (30 m resolution) value for mangrove habitat by dividing the catch per subregion by the number of mangrove pixels. For the protect mangrove strategy, the denominator was the existing baseline mangrove footprint in each subregion (for example, the area that might be lost without protection). For the restore mangrove strategy, the denominator was the extent of degraded or cleared mangrove in each subregion (Designing mangrove strategies section above). In addition, the optimization prioritizes hexagons with the largest return in benefits for the smallest investment in the area of habitat protected or restored. While this approach incorporates area as a proxy for cost, it does have limitations, especially for the lobster model. We found that the results prioritized small patches of mangroves linked to subregions with high catch values and deprioritized large sections of mangroves (especially south of Belize City) that in aggregate provide substantial nursery habitat but provide fewer benefits per unit ha of mangroves.

The assumptions and limitations of the population model related to natural mortality, the harvest function and selectivity and market value are discussed at length elsewhere^25,77^. Most relevant to this analysis are the simplifying assumptions that all mangrove functions equally well as nursery habitat for lobster and that the availability of suitable nursery habitat is important for recruitment to the local population. Several studies indicate that lack of suitable nursery habitat can decouple the relationship between larval supply and recruitment to lobster populations across the Caribbean^84,85^. However, recruitment to mangroves and juvenile survivorship varies based on multiple factors such as oceanographic currents and the regional distribution of marine protected areas that are beyond the scope of our modelling framework^84^. In addition, we do not differentiate between taxa, density or other characteristics of mangrove patches. Juvenile lobster recruits to mangrove proportionally to its availability within each subregion. The model does not account for recruitment hotspots or source/sink larval dynamics that may exist. Similarly, the lobster population responds to changes in the area of mangrove habitat, not other characteristics such as change in mangrove density or patchiness. We assume the mangrove in a given subregion ultimately has the same per pixel value for contribution to lobster catch because it is extracted from a single marginal catch value. Factors that govern the catch for a given subregion include availability of adult habitat, emigration from other subregions and fishery behaviour. The patterns influencing prioritization within a given subregion are driven by the extent of mangrove within a SDU. Furthermore, the population growth parameters are nationwide, not region specific, as there were not sufficient data for estimation of region-specific parameters. Habitat dependencies are obligatory, such that lobster do not have the option to seek out acceptable substitutes but instead are constrained to depend on mangroves, seagrass and corals as defined in the model. In spite of these assumptions related to habitat, comparisons between modelled results and empirical information at both country and subregion scales indicate good agreement. Estimates of annual catch fall within the range of empirical data for the same time period and those subregions in Belize with highest modelled catch align with places where the Belize Fisheries Department reports the highest catch^25,77^.

#### Tourism

To quantify the contribution of mangroves to tourism in Belize, we estimated the spatial distribution of visitation across the entire Mesoamerican Reef region following (and extending) the social media-based approach in the InVEST Recreation model^25,64^. The approach uses geotagged posts to Flickr and Twitter as a proxy for visitation. Posts to these social media platforms have been shown to be well correlated with empirical visitation numbers^86–89^ and useful for approximating visitation across large spatial scales^89–91^.

We estimated tourism across the coastal zone of Mexico, Belize, Guatemala and Honduras (roughly 75 km offshore to 50 km inland) from the northern point of the Yucatan Peninsula to the northern coast of Honduras. The area of interest extends beyond Belize because we originally developed this model to inform a Mesoamerican Reef-wide climate adaptation project. First, we created spatially explicit estimates of absolute visitation using Flickr, Twitter and national tourism statistics. We estimated tourism to 5 km grid cells by apportioning the total number of international overnight and cruise ship tourists who visited each country in 2017 (1.44 million visitors to Belize, as reported by the Belize Tourism Board), according to the proportion of Flickr photo user days (the number of unique social media users who posted each day) and Twitter user days posted within each grid cell. These proportions were based on the average annual number of user days posted to each platform (Flickr 2005–2018, Twitter 2012–2018). Flickr images were retrieved from 1–2 January 2019 by querying the Flickr API, while Tweets were retrieved in real time from Twitter’s ‘statuses/filter’ streaming API. We queried each platform for all geolocated posts that users shared during the study period from within the study area. We assigned equal weight to photo user days and Twitter user days because we had no reason to believe that one was more closely related to tourism in the region than the other^89,92,93^.

We then related the estimated number of visitors to underlying natural, cultural and infrastructure features and to average climatic conditions to gain an understanding of visitors’ preferences using multiple linear regression^25,87,94^. We selected features that we expected to drive patterns in visitation and which matched inputs into other ecosystem service models. We also included downscaled climatic data to capture the potential influence of spatial variation in the average annual temperature, number of hot days (>35 °C), and precipitation^95^. Additionally, because we modelled visitation across the entire Mesoamerican Reef region, we included a categorical variable for country and a controlling variable for the size of the grid cell. Variables were rescaled to fall between 0 and 1, then checked for multicollinearity (all pairwise correlations were below 0.6 and all variance inflation factor (VIF) values were below 2). In the interest of model parsimony, we did not include any interaction terms. All variables were significant at *α* = 0.05, so we did not do further model selection. Mangroves had a significantly positive effect on visitation (coefficient = 1.958, *p* value < 0.0001). The full set of features are described in Supplementary Table 1. Together, they explained 45% of the variability in visitation across the region (adjusted *R*^2^ = 0.454; Supplementary Table 6).

Using the relationship between tourism and mangroves that we quantified in the regression model, we predicted how visitation might change with mangrove restoration and protection. Because we held all other variables constant (that is, natural, cultural and infrastructure features, climatic conditions; Supplementary Table 6), these predicted values represent the marginal effect of mangrove on tourism. We then calculated the difference between the number of tourists predicted under the restoration strategy and the number of tourists predicted under the baseline (using the fitted values of the regression) for each grid cell. We repeated this analysis for the protect mangrove strategy, which was modelled by assuming that all mangroves were lost, if not protected.

The tourism model makes a few key assumptions. First, we assume that Twitter and Flickr users choose to travel in ways that are representative of all visitors to the region. While we recognize that each of these datasets probably represents a biased sample of visitors, we believe that by including Twitter, in addition to Flickr, we are mitigating some of these biases^92^. Related, we chose to focus on international tourists for this analysis, due to difficulties in finding reliable empirical data on domestic tourism. Further, the preference model assumes that visitors’ preferences are static throughout time. An implication of this is that we are assuming tourists will place the same value on healthy mangroves in the future as they do today. We additionally assume that visitors’ decisions about where to travel within the region, as quantified in the preference model, explain their decisions to travel to the region. Lastly, by applying a model that was developed for the entire Mesoamerican Reef, we are assuming that the influence of mangroves on tourism is comparable across the region. This assumption is in line with our previous work on tourism and mangroves in Belize alone^25^.

### Reporting summary

Further information on research design is available in the Nature Portfolio Reporting Summary linked to this article.

## Supplementary information

Supplementary Information Supplementary Tables 1–6.2

Reporting Summary

## Data Availability

The ecosystem service and optimization data are available through Figshare at 10.6084/m9.figshare.22123634 ref. ^96^.

## Code availability

Links for downloading ROOT and the InVEST open source software are available at naturalcapitalproject.stanford.edu. The source code is available at https://github.com/natcap/invest.
