# Supplementary material for: Evidence-based target setting informs blue carbon strategies for nationally determined contributions
Source: Nat Ecol Evol. 2023 Jun 1;7(7):1045–59. doi: 10.1038/s41559-023-02081-1 (PMC10333125; doi:10.1038/s41559-023-02081-1)
Supplement: Supplementary file 1 — Supplementary Tables 1–6.2 [file 41559_2023_2081_MOESM1_ESM.pdf]

nature ecology & evolution

Article https://doi.org/10.1038/s41559-023-02081-1

Evidence-based target setting informs blue carbon strategies for nationally determined contributions

In the format provided by the authors and unedited

Supplementary Information

Table of Contents

Supplementary Table 1. Data sources for mapping protection and restoration strategies and modeling ecosystem services 2

Supplementary Table 2. Range of potential target areas for blue carbon strategies. 6

Supplementary Table 3. Carbon storage estimates assuming soil carbon stocks to 0.5 m depth ... 7

Supplementary Table 4. Variables in the InVEST Coastal Vulnerability model and ranking system. 8

Supplementary Table 5. Habitat specific ranks and protective distances 9

Supplementary Table 6. Variables used in the tourism model 10

Supplementary Table 1. Data sources for mapping protection and restoration strategies and modeling ecosystem services

Model	Model Input		Year	Extent	Resolution	Source
Coastal risk reduction; Spiny lobster fishery; Tourism	Natural Habitats	Coral Reef (barrier and fringing)	2020	Meso-American Reef	30m	Developed using spatial predictive modeling (boosted regression trees). The models were calibrated using empirical cover % cover from reef surveys data (N=500, HRI) and geospatial datasets (bathymetry [30m] [TNC], wave power [ERA5], and modeled Total Suspended Sediment [derived from SDR & WQ models]) as predictors. Once calibrated, coral % cover was geographically extrapolated using values from the geospatial datasets.
Coastal risk reduction; Spiny lobster fishery	Natural Habitats	Seagrass	1997	Belize (offshore)	1:110,000	Developed as a collaboration between the Coastal Zone Management Project, the University of Exeter, the University of Newcastle and Coral Caye Conservation. This effort mapped offshore seagrass habitat in Belize.
Coastal risk reduction; Spiny lobster fishery	Natural Habitats	Seagrass	2007	Caribbean-wide		University of British Columbia led regional-scale seagrass habitat mapping in the Wider Caribbean Region using Landsat sensors. This effort was used to supplement the 1997 local dataset and fill in the nearshore seagrass.
Coastal risk reduction; Spiny lobster fishery; Tourism	Natural Habitats	Coastal Forest (non-mangrove lowland broadleaf multispecies forest)	2017	Belize	100m	Landsat 8 imagery classified based on UNESCO system for Central American Ecosystems. This product is an updated version of earlier national LULC maps in 2004, 2011, and 2015.
Coastal risk reduction; Spiny lobster fishery;	Natural Habitats	Mangrove (Current extent)	2019	Belize	30m	Landsat 5, 7 & 8 imagery classified by Cherrington et al. 2020. This dataset represents Belize's national mangrove cover in 2019, based on satellite-based mapping of Belize's mangroves for 1980, 1989, 1994, 2000, 2004, and

Tourism						2010, and based on the earlier work of Simon Zisman (1998).
Coastal risk reduction; Spiny lobster fishery; Tourism	Natural Habitats	Mangrove (Cleared or Degraded)	2019	Belize	30m	Landsat 5, 7 & 8 imagery classified by Cherrington et al. 2020. Mangrove cleared in recent years has been recorded as part of the national mangrove cover mapping effort described above.
Coastal risk reduction; Spiny lobster fishery; Tourism	Natural Habitats	Mangrove (Cleared or Degraded)	2010	Belize	30m	This dataset was developed using remote sensing of satellite imagery in collaboration between the Mesoamerican Reef program of the World Wildlife Fund and the Regional Visualization & Monitoring System (SERVIR) initiative jointly implemented by the Water Center for the Humid Tropics of Latin America and the Caribbean (CATHALAC), NASA, USAID and other partners. The goals of the dataset were to identify (i) fragmented mangrove ecosystems, (ii) mangroves at risk of fragmentation, and (iii) the resilient mangroves.
Coastal risk reduction	Relief	Digital Elevation Model (90 m)	2014	Global	90 m	Shuttle Radar Topography Mission (SRTM) v4
Coastal risk reduction	Wave & Wind Exposure		2005-2010	Global	50 km	National Oceanographic and Atmospheric Administration WaveWatch III
Coastal risk reduction; Tourism	Shoreline Type	Coastal Geomorphology	2012	Belize	vector	Digitized from Google Earth aerial imagery by the Natural Capital Project and WWF.
Coastal risk reduction	Surge Potential	Continental Shelf	2005	Global	vector	Continental Margins Ecosystem (COMARGE) effort in conjunction with the Census of Marine Life
Tourism	Wildlife	Wildlife-based tourism destinations	2019	Meso-American Reef	vector	Habitat or key tourism destinations for flamingos, manatees, sea turtles, sharks, and whale sharks. Data are a

						variety of observations, habitat, and tourism destinations identified by WWF and compiled by the Natural Capital Project.
Tourism	Archeological sites	Major archeological sites	2019	Meso-American Reef	vector	Major archeological destinations in the region, created with data from OpenStreetMap and updated by the Natural Capital Project.
Tourism	Development	LULC map	2017<br>2010<br>2014<br>2013	Belize<br>Guatemala<br>Honduras<br>Mexico	vector<br>1:50,000<br>5 m	A LULC map for the Mesoamerican Reef (MAR) region was created based on four sources: Meerman J.C. 2017 Map of the Ecosystems of Belize version 2017, accessed at: http://www.biodiversity.bz/ (Belize); DIGEGR 2015 Mapa de cobertura vegetal y use de la tierra, a escala 1:50,000 de le republica de Guatemala ano 2010, Memoria técnica y descripción de resultados, June 2015, Guatemala, pp 215 (Guatemala); Mapa de Cobertura Forestal 2014 (Forest Cover map 2014), from ICF Forest Sector Geoportal (Honduras); and SEMARNAT 2014 Inventario estatal forestal y de suelos - Quintana Roo 2013 (Mexico).The tourism analysis used the “developed” class in the LULC map to create a footprint of current development.
Tourism	Roads		2019	Meso-American Reef	vector	Major roads in the MesoAmerican Reef region. Created by the Natural Capital Project by selecting “primary”, “secondary”, and “trunk” roads from data from mapcruzin.com, and then incorporating feedback from local partners.
Tourism	Airports / Ports		2019	Meso-American Reef	vector	Primary airports and sea ports in the region. Created by the Natural Capital Project with data from Natural Earth (airports), Mexico port layer (ports), humdata.org (ports), and contributions from project partners.
Tourism	Climate	Temperature		Global	0.25 degrees	Average annual temperature. Baseline and 2050s RCP8.5

						projections from Columbia University
Touris m		Extreme heat days		Meso-American Reef	0.25 degrees	Average number of days above 35C, annually. Baseline and 2050s RCP8.5 projections from Columbia University.
Touris m		Precipitation		Global	0.25 degrees	Total annual precipitation. Baseline and 2050s RCP8.5 projections from Columbia University.

Supplementary Table 2. Range of potential target areas for blue carbon strategies.

Range of potential target areas (in thousands of hectares (ha)) for investments in mangrove protection and restoration strategies. The largest target for protection and restoration represents the full opportunity area.

Mangrove protection (thousands of ha)		Mangrove restoration (thousands of ha)
5		1
10		5
20		10
25		13
64		

Supplementary Table 3. Carbon storage estimates assuming soil carbon stocks to 0.5 m depth

We also estimated carbon stored assuming soil carbon stocks to 1 m. The 1 m results for the full opportunity areas (protection = 64,000 ha; restoration = 13,000 ha) and selected targets (protection = 12,000 ha; restoration = 4,000 ha) are reported in the text of the main manuscript.

Strategy	Target (ha)	Carbon stored (0.5 m depth)	
		MMT C	MMT CO_2e
protection	5,000	2.69	9.87
protection	10,000	4.39	16.08
protection	12,000	5.05	18.52
protection	20,000	8.77	32.17
protection	25,000	10.97	40.21
protection	64,000	28.07	102.93
restoration	1,000	0.13	0.47
restoration	4,000	0.48	1.77
restoration	5,000	0.64	2.35
restoration	10,000	1.28	4.71
restoration	13,000	1.67	6.10

Supplementary Table 4. Variables in the InVEST Coastal Vulnerability model and ranking system.

Ranks for the relief, wave exposure and surge potential are calculated from the full distribution of values for all 250 m shoreline segments across Belize. These data were adapted for Belize from Table 1 in Silver et al. 2019, Supplementary Table 1 in Arkema et al. 2013, and Table 4.1 in Sharp et al 2020.

Rank<br><br>Variable	Very low<br>1	Low<br>2	Moderate<br>3	High<br>4	Very High<br>5
Natural habitats	Coral reef (Barrier and Fringing), Coastal Forest, Mangrove			Seagrass	
Shoreline type	Large seawall	Small seawall	Riprap	Mud	Sand
Relief	1rst quantile	2nd quantile	3rd quantile	4th quantile	5th quantile
Wave exposure	1rst quantile	2nd quantile	3rd quantile	4th quantile	5th quantile
Wind exposure	1rst quantile	2nd quantile	3rd quantile	4th quantile	5th quantile
Surge potential	1rst quantile	2nd quantile	3rd quantile	4th quantile	5th quantile

Supplementary Table 5. Habitat specific ranks and protective distances

Habitat	Rank	Protective Distance
Coral Reef (fringing)	1	2,000m
Coral Reef (barrier)	1	35,000m
Seagrass	4	500m
Coastal Forest	1	2,000m
Mangrove Forest	1	2,000m

Supplementary Table 6. Variables used in the tourism model

Variables used in the tourism model, their descriptions, and how each variable was transformed prior to inclusion in the generalized linear model described in the methods (adjusted R2=0.45, n=7793, degrees of freedom=7792). We also present the estimated coefficients, standard errors, t-values, and p-values for each coefficient. OSM = Open Street Map https://www.openstreetmap.org/copyright

Variable	Description	Variable treatment	Coefficient	Standard error	t-value	p-value
Visitors (Response)	Estimated annual visitors per grid cell, based on Flickr, Twitter, and national tourism statistics.	Log transformed	NA	NA	NA	NA
Intercept	NA	NA	1.054	0.242	4.350	1.38e-05
Mangroves	Proportion of the grid cell covered by healthy mangrove	NA	1.959	0.362	5.417	6.26e-08
Coral	Proportion of the grid cell covered by healthy coral	NA	4.615	0.376	12.284	< 2e-16
Beach	Sandy beaches	Presence/Absence	1.870	0.103	18.110	< 2e-16
Coastal forest	Proportion of the grid cell covered by coastal forest (as defined Supp Table 2)	NA	0.846	0.079	10.728	< 2e-16
Wildlife	Key habitat or tourism destination for flamingos, manatees, sharks, whale sharks, sea turtles. Created in collaboration with local partners	Presence/Absence	0.492	0.080	6.124	9.57e-10

Archeological sites	Key archeological sites in the region, from OSM	Presence/Absence	2.691	0.438	6.149	8.20e-10
Development	“Developed” land class from national LULC raster	Presence/Absence	1.877	0.034	22.444	< 2e-16
Roads	Major local roads, from OSM	Presence/Absence	1.805	0.089	20.355	< 2e-16
Airports / Ports	Minimum distance to the nearest major airport or port, compiled from OSM and local sources	Rescaled to fall between 0 and 1	- 4.467	0.146	-30.654	< 2e-16
Temperature	Average annual temperature	Rescaled to fall between 0 and 1. Quadratic	Temp: 2.586<br>$Temp^2$: -3.103	0.661<br>0.589	3.915<br>-5.265	9.13e-05<br>1.44e-07
Hot Days	Number of days/year > 35C	Rescaled to fall between 0 and 1	- 0.417	0.174	-2.391	0.0168
Precipitation	Total annual precipitation	Rescaled to fall between 0 and 1	0.689	0.176	3.905	9.49e-05
Country	Country in which a grid cell occurred	Factor, Belize is the baseline	Guatemala: -1.891<br>Honduras: -0.889<br>Mexico: -0.246	0.131<br>0.070<br>0.089	-14.405<br>-12.736<br>-2.765	< 2e-16<br>< 2e-16<br>0.006
Cell area	Control for variable sized cells	Rescaled to fall between 0 and 1	1.829	0.099	18.403	< 2e-16
