# Supplementary material for: Evidence-based target setting informs blue carbon strategies for nationally determined contributions
Source: Nat Ecol Evol. 2023 Jun 1;7(7):1045–59. doi: 10.1038/s41559-023-02081-1 (PMC10333125; doi:10.1038/s41559-023-02081-1)
Supplement: Supplementary file 2 — Reporting Summary [file 41559_2023_2081_MOESM2_ESM.pdf]

nature portfolio

Corresponding author(s): Katie Arkema

Last updated by author(s): 3/29/2023

Reporting Summary

Nature Portfolio wishes to improve the reproducibility of the work that we publish. This form provides structure for consistency and transparency in reporting. For further information on Nature Portfolio policies, see our Editorial Policies and the Editorial Policy Checklist.

Statistics

For all statistical analyses, confirm that the following items are present in the figure legend, table legend, main text, or Methods section.

n/a	Confirmed	
☒	☐	The exact sample size (*n*) for each experimental group/condition, given as a discrete number and unit of measurement
☒	☐	A statement on whether measurements were taken from distinct samples or whether the same sample was measured repeatedly
☒	☐	The statistical test(s) used AND whether they are one- or two-sided<br>*Only common tests should be described solely by name; describe more complex techniques in the Methods section.*
☒	☐	A description of all covariates tested
☒	☐	A description of any assumptions or corrections, such as tests of normality and adjustment for multiple comparisons
☒	☐	A full description of the statistical parameters including central tendency (e.g. means) or other basic estimates (e.g. regression coefficient) AND variation (e.g. standard deviation) or associated estimates of uncertainty (e.g. confidence intervals)
☒	☐	For null hypothesis testing, the test statistic (e.g. *F*, *t*, *r*) with confidence intervals, effect sizes, degrees of freedom and *P* value noted<br>*Give P values as exact values whenever suitable.*
☒	☐	For Bayesian analysis, information on the choice of priors and Markov chain Monte Carlo settings
☒	☐	For hierarchical and complex designs, identification of the appropriate level for tests and full reporting of outcomes
☒	☐	Estimates of effect sizes (e.g. Cohen's *d*, Pearson's *r*), indicating how they were calculated

Our web collection on statistics for biologists contains articles on many of the points above.

Software and code

Policy information about availability of computer code

Data collection	ArcGIS was used to pre-process, map, and visually compare spatial data used and produced by this study.
Data analysis	Links for downloading ROOT and the InVEST open-source software are available at naturalcapitalproject@stanford.edu. The source code is available at https://github.com/natcap/invest.

For manuscripts utilizing custom algorithms or software that are central to the research but not yet described in published literature, software must be made available to editors and reviewers. We strongly encourage code deposition in a community repository (e.g. GitHub). See the Nature Portfolio guidelines for submitting code & software for further information.

Data

Policy information about availability of data

All manuscripts must include a data availability statement. This statement should provide the following information, where applicable:

- Accession codes, unique identifiers, or web links for publicly available datasets
- A description of any restrictions on data availability
- For clinical datasets or third party data, please ensure that the statement adheres to our policy

The ecosystem service and optimization data are available through Figshare at the following link: 10.6084/m9.figshare.22123634.

Field-specific reporting

Please select the one below that is the best fit for your research. If you are not sure, read the appropriate sections before making your selection.

☐ Life sciences ☐ Behavioural & social sciences ☒ Ecological, evolutionary & environmental sciences

For a reference copy of the document with all sections, see nature.com/documents/nr-reporting-summary-flat.pdf

Ecological, evolutionary & environmental sciences study design

All studies must disclose on these points even when the disclosure is negative.

Study description	To inform Belize's update to their Nationally Determined Contributions (NDCs), we quantified carbon storage and sequestration using existing field estimates, and coastal risk reduction, tourism, and fisheries co-benefits by modeling ecosystem services provided by mangroves currently and under two blue carbon strategies (conserve mangroves and restore mangroves). To identify priority locations for investing in blue carbon strategies, we optimized co-benefits. The results were used by the Belize Blue Carbon Working Group and other government agencies to set the targets for mangrove protection and restoration in Belize's update to it's NDCs, submitted to the UNFCCC in fall of 2021.
Research sample	Our modeling study takes place in Belize. We quantified ecosystem services across the country and calculated the expected marginal change between baseline service provisioning and services provided under the two blue carbon strategies in each 30 m grid cell. We then optimized for several ecosystem services at a resolution of 1000 ha hexagons.
Sampling strategy	We included the entire coastal zone of Belize in our analysis, so no sampling was required.
Data collection	We collected existing spatial data from a variety of academic, NGO, and governmental institutions. We compiled original versions of the source data and pre-processed data on google drive in structured data folders.
Timing and spatial scale	The spatial scale of the study was the entire coastal region of Belize. This was not an experimental study. The source data for the models range from 2007-2020.
Data exclusions	No data were excluded.
Reproducibility	We expect the analytical portions of this study (ecosystem service modeling and optimization) to be reproducible. To some extent, the policy aspects of the study could be "reproducible" from an adaptive management perspective for future iterations of the NDCs. How these science-policy components play out in the future will vary depending on the decision-making needs and context.
Randomization	This study is not an experimental study
Blinding	This study is not an experimental study

Did the study involve field work? ☐ Yes ☒ No

Reporting for specific materials, systems and methods

We require information from authors about some types of materials, experimental systems and methods used in many studies. Here, indicate whether each material, system or method listed is relevant to your study. If you are not sure if a list item applies to your research, read the appropriate section before selecting a response.

Materials & experimental systems

n/a	Involved in the study	
☒	☐	Antibodies
☒	☐	Eukaryotic cell lines
☒	☐	Palaeontology and archaeology
☒	☐	Animals and other organisms
☒	☐	Human research participants
☒	☐	Clinical data
☒	☐	Dual use research of concern

Methods

n/a	Involved in the study	
☒	☐	ChIP-seq
☒	☐	Flow cytometry
☒	☐	MRI-based neuroimaging
